# Real-time continuous glucose monitoring in preterm infants (REACT): an international, open-label, randomised controlled trial

**DOI:** 10.1016/S2352-4642(20)30367-9

**Published:** 2021-04

**Authors:** Kathryn Beardsall, Lynn Thomson, Catherine Guy, Isabel Iglesias-Platas, Mirjam M van Weissenbruch, Simon Bond, Annabel Allison, Sungwook Kim, Stavros Petrou, Beatrice Pantaleo, Roman Hovorka, David Dunger, REACT collaborative, Zoltan Molnar, Sheula Barlow, Sharon Baugh, Kathryn Johnson, Lindsay Uryn, Collette Spencer, Maria Hubbard, Sateeshkumar Somisetty, Olaitan Adesiyan, Jogesh Kapadia, Yvonne Millar, Kalyana Gurusamy, Lindsay Bibb, Kathryn Jones, Richard Heaver, Priya Muthukumar, Amy Nichols, Mark Johnson, Jenny Pond, Philippa Crowley, Christie Mellish, Divyen D Shah, Mercy Abraham, Presillina Vincent, Suma Anil kumar, Angelina Iringan, Barbara Aninakwa, R A Dalangin-Chalmers, Annemieke de Lange

**Affiliations:** a Department of Paediatrics, University of Cambridge, Cambridge, UK; b Wellcome Trust MRC Institute of Metabolic Science, University of Cambridge, Cambridge, UK; c Neonatal Unit, Cambridge University Hospitals NHS Foundation Trust, Cambridge, UK; d Cambridge Clinical Trials Unit, Cambridge University Hospitals NHS Foundation Trust, Cambridge, UK; e Neonatal Unit, Institut de Recerca, Sant Joan de Déu, Barcelona, Catalonia, Spain; f Department of Paediatrics, VU University Medical Center, Amsterdam, Netherlands; g Warwick Clinical Trials Unit, The University of Warwick, Coventry, UK; h Nuffield Department of Primary Care Health Sciences, University of Oxford Radcliffe Observatory Quarter, University of Oxford, Oxford, UK

## Abstract

**Background:**

Hyperglycaemia and hypoglycaemia are common in preterm infants and have been associated with increased risk of mortality and morbidity. Interventions to reduce risk associated with these exposures are particularly challenging due to the infrequent measurement of blood glucose concentrations, with the potential of causing more harm instead of improving outcomes for these infants. Continuous glucose monitoring (CGM) is widely used in adults and children with diabetes to improve glucose control, but has not been approved for use in neonates. The REACT trial aimed to evaluate the efficacy and safety of CGM in preterm infants requiring intensive care.

**Methods:**

This international, open-label, randomised controlled trial was done in 13 neonatal intensive care units in the UK, Spain, and the Netherlands. Infants were included if they were within 24 h of birth, had a birthweight of 1200 g or less, had a gestational age up to 33 weeks plus 6 days, and had parental written informed consent. Infants were randomly assigned (1:1) to real-time CGM or standard care (with masked CGM for comparison) using a central web randomisation system, stratified by recruiting centre and gestational age (<26 or ≥26 weeks). The primary efficacy outcome was the proportion of time sensor glucose concentration was 2·6–10 mmol/L for the first week of life. Safety outcomes related to hypoglycaemia (glucose concentrations <2·6 mmol/L) in the first 7 days of life. All outcomes were assessed on the basis of intention to treat in the full analysis set with available data. The study is registered with the International Standard Randomised Control Trials Registry, ISRCTN12793535.

**Findings:**

Between July 4, 2016, and Jan 27, 2019, 182 infants were enrolled, 180 of whom were randomly assigned (85 to real-time CGM, 95 to standard care). 70 infants in the real-time CGM intervention group and 85 in the standard care group had CGM data and were included in the primary analysis. Compared with infants in the standard care group, infants managed using CGM had more time in the 2·6–10 mmol/L glucose concentration target range (mean proportion of time 84% [SD 22] *vs* 94% [11]; adjusted mean difference 8·9% [95% CI 3·4–14·4]), equivalent to 13 h (95% CI 5–21). More infants in the standard care group were exposed to at least one episode of sensor glucose concentration of less than 2·6 mmol/L for more than 1 h than those in the intervention group (13 [15%] of 85 *vs* four [6%] of 70). There were no serious adverse events related to the use of the device or episodes of infection.

**Interpretation:**

Real-time CGM can reduce exposure to prolonged or severe hyperglycaemia and hypoglycaemia. Further studies using CGM are required to determine optimal glucose targets, strategies to obtain them, and the potential effect on long-term health outcomes.

**Funding:**

National Institute for Health Research Efficacy and Mechanisms Evaluation Programme.

## Introduction

Hyperglycaemia, hypoglycaemia, and glycaemic instability are common in preterm infants and have been associated with increased risk of mortality and morbidity.^1^, ^2^, ^3^, ^4^, ^5^ Hyperglycaemia can lead to acute problems of osmotic diuresis and metabolic acidosis, and has been associated with increased risk of intraventricular haemorrhage,^5^ patent ductus arteriosus, retinopathy of prematurity,^1^ necrotising enterocolitis,^3^ and a reduction in white matter.^6^ Attempts to reduce risks associated with hyperglycaemia can increase the risk of hypoglycaemia,^7^ which has been associated with poor developmental outcomes.^4^

However, maintaining normoglycaemia in preterm infants is challenging due to the highly variable combination of insulin resistance and insulin deficiency, as well as low energy reserves in individual babies. The current practice of infrequent, intermittent blood glucose measurement adds to the difficulties of avoiding extremes of blood glucose concentrations.^8^ Furthermore, studies using masked subcutaneous continuous glucose monitoring (CGM) have shown extended episodes of both hyperglycaemia and hypoglycaemia that were clinically silent but were associated with worse developmental outcomes in childhood.^9^, ^10^

**Research in context**

**Evidence before this study**

Both hyperglycaemia and hypoglycaemia are common in preterm infants and are associated with mortality and morbidity. Management is challenging because high energy and nutritional requirements are needed to support growth, and variable insulin sensitivity makes infants at risk from both hyperglycaemia and hypoglycaemia. Hyperglycaemia can lead to acute problems of osmotic diuresis and metabolic acidosis, and has been associated with increased risk of intraventricular haemorrhage, retinopathy of prematurity, and necrotising enterocolitis. Continuous glucose monitoring (CGM) has shown that periods of clinically silent hypoglycaemia are associated with later impaired developmental outcomes. The use of CGM in vulnerable preterm infants could allow earlier detection and potentially prevention of exposure to extreme glucose concentrations. Real-time CGM has been used in adult and paediatric intensive care to optimise glucose control, and preliminary data suggest it is feasible in preterm infants. We searched PubMed, Cochrane Library, and clinical trials databases on Oct 31, 2014, using the search terms “continuous glucose monitoring”, “preterm”, “hyperglycemia”, and “hypoglycemia” for clinical trials published between 1966, and Oct 31, 2014, with no language restrictions. There were no randomised controlled trials of real-time CGM in preterm infants; however, an updated search in October, 2020, identified four small, single-centre, randomised studies exploring the feasibility of using CGM to target glucose control, but this has not been formally evaluated as part of a multicentre trial.

**Added value of this study**

To our knowledge, this is the first multicentre trial to evaluate the efficacy, safety, and use of CGM in preterm infants by allowing clinicians to use CGM data to optimise nutritional delivery as well as providing guidance on using insulin. We show that real-time CGM can improve the targeting of glucose control in preterm infants, with increased time in the target glucose concentration range of 2·6–10 mmol/L compared with standard care. This was achieved by reducing the number of infants exposed to prolonged or severe hyperglycaemia and episodes of hypoglycaemia lasting more than 1 h. The exploratory finding that the rate of necrotising enterocolitis was higher in the standard care group than in the real-time CGM group warrants further investigation. Although the real-time CGM device was not designed for use in newborn infants, there were no reported serious adverse effects.

**Implications of all the available evidence**

This study supports the clinical use of CGM in preterm infants to optimise nutritional delivery alongside improving glucose monitoring and management. CGM can provide more targeted glucose control with less invasive blood glucose testing. Further studies will be important to evaluate the longer-term effect on clinical outcomes.

Therefore, improving early glucose control might be an important modifiable risk factor for outcomes in preterm infants. The development of real-time CGM, which provides continuous glucose data and identifies early trends in glucose concentrations can inform clinical decisions, and has been successfully used in adult^11^ and paediatric intensive care.^12^, ^13^, ^14^ Preliminary data suggest it is feasible in preterm infants^10^, ^15^, ^16^, ^17^ and could allow earlier detection and potentially prevention of exposure to extreme glucose concentrations.^17^, ^18^ We aimed to formally evaluate the use of real-time CGM to guide the clinical management of glycaemic control and use of insulin in preterm infants.

## Methods

### Study design and participants

This was an international, open-label, parallel-group, randomised controlled trial done in 13 neonatal intensive care units in the UK, Spain, and the Netherlands. Infants were included if they were within 24 h of birth, had a birthweight of 1200 g or less, had a gestational age up to 33 weeks plus 6 days, and had parental written informed consent. Exclusion criteria included any lethal congenital malformations or congenital metabolic disorders. The trial was reviewed and approved in the UK by the Health Research Authority (IRAS ID 168042), Research Ethics Committee (reference 15/EE/0158), and the Medicines and Healthcare products Regulatory Agency (reference CI/2016/0011), as well as by all local regulatory boards. International regulatory approvals were obtained for non-UK sites. The study was coordinated and monitored by the National Institute for Health Research Cambridge Clinical Trials Unit (Cambridge, UK) in accordance with international guidelines. An independent data monitoring and ethics committee and a trial steering committee were appointed and reviewed the data according to their formal charters. The protocol has been published elsewhere.^19^

### Randomisation

Babies were randomly assigned (1:1) within 24 h of birth to receive either the intervention with real-time CGM or standard care until 7 days of age.^19^ Randomisation was done using a central web randomisation system, Trans European Network ALEA, using blocks of random size (four, six, eight), stratifying by recruiting centre and gestational age (<26 or ≥26 weeks). Masking of the study intervention was not feasible.

### Procedures

All infants had an Enlite glucose sensor (Medtronic, Northridge, CA, USA) inserted subcutaneously into the thigh. Sensors were inserted by hand, not using the standard insertion device, to ensure that they were inserted into the subcutaneous tissue. A blood glucose sample was required every 12 h for calibration and each neonatal intensive care unit was provided with Nova StatStrip meters (Nova Biomedical, Waltham, MA, USA) to provide consistency in this measure across sites. Clinical outcome data were collected until 36 weeks' corrected gestational age. The real-time CGM system comprised an Enlite sensor and a Guardian 2 Link transmitter (Medtronic, Northridge, CA, USA), linking to a 640G monitor (MiniMed 640G, Medtronic, Northridge, CA, USA). The system was used solely to monitor the glucose concentrations as described previously.^20^ Glucose concentrations outside the range 2·2–22·2 mmol/L (40–400 mg/dL) are recorded by the monitor as less than 2·2 mmol/L (40 mg/dL), or more than 22·2 mmol/L (400 mg/dL). The system is not licensed for use in neonatal intensive care.

For infants assigned to the intervention group, the real-time CGM data were available to view by the clinical team during the first week of life. Clinical staff were advised to read and record the sensor glucose data hourly and were provided with a specifically designed guideline to aid the management of glucose control on the basis of the CGM data. This included modification of the rate of dextrose infusion or use of supplementary insulin. This guidance was developed during the REACT feasibility study,^18^ and provides advice based on trends as well as absolute glucose concentrations (appendix p 2). The clinical decision making, including changes in dextrose and insulin infusion, was based primarily on the real-time CGM data but the guideline advised checking blood glucose concentrations when there were rapid changes in CGM values, or if CGM values fell to less than 4 mmol/L to ensure safety. Considering the feedback from the feasibility study, the threshold and prediction alarms on the real-time CGM devices were switched off.^18^ All other aspects of clinical care were at the discretion of local staff.

Infants assigned to the control group had glucose control monitored and managed according to local standard clinical practice using intermittently sampled blood glucose concentrations. All aspects of clinical care were at the discretion of local staff. The real-time CGM device collected glucose data continuously but the values were masked to the clinical team (in an opaque bag with a tamper proof seal). Calibration was required every 12 h, and compliance was documented with a log that recorded timings of the tamper tag being broken and resealed.

### Outcomes

The primary efficacy outcome was the proportion of time that sensor glucose concentrations were in the target range of 2·6–10 mmol/L up to 7 days of life, compared between study groups. This was selected as the most widely accepted international target for glucose control in this population,^21^ outside of which clinical intervention would be put in place due to associated adverse effects. Secondary efficacy outcomes were: the proportion of time sensor glucose concentrations were in the target range of 4–8 mmol/L; overall mean sensor glucose concentration, sensor glucose concentration variability (assessed by within-patient standard deviation), and proportion of time that sensor glucose concentrations were in the severe hyperglycaemic range (>15 mmol/L). Safety outcomes were incidence of hypoglycaemia (any recorded blood glucose concentration of 2·2–2·6 mmol/L or any continuous episode of sensor glucose concentration of <2·6 mmol/L for >1 h) and severe hypoglycaemia (any recorded blood glucose ≤2·2 mmol/L).

Predefined exploratory analyses were done on clinical outcomes: mortality before 36 weeks' corrected gestational age; retinopathy of prematurity (maximum grade across all examinations); bronchopulmonary dysplasia (need for supplemental oxygen or respiratory support at 36 weeks' corrected gestational age); infection (microbiologically confirmed or clinically suspected late onset invasive infection from trial entry until hospital discharge); necrotising enterocolitis (requiring surgical intervention including peritoneal drainage or causing death); patent ductus arteriosus (requiring medical or surgical treatment); intracerebral pathology before discharge; growth at the end of week 1 and at 36 weeks' corrected gestational age; nutritional intake in week 1; and use of insulin in weeks 1 and 2.^19^ The use of real-time CGM was assessed using a questionnaire, which was completed anonymously by staff caring for an infant on days 3 and 7 (to provide an unbiased sample), and a similar parent questionnaire completed on day 7. A prospective economic evaluation was done as an exploratory analysis and will be reported elsewhere. All outcomes were centrally assessed.

### Statistical analysis

The sample size was based on data from the REACT feasibility study and historical control data,^10^, ^18^ which conservatively assumed that the SD of the primary endpoint would be 22%. Initially, a sample size of 200 participants was calculated to be needed to detect a treatment effect of a 10% increase in the mean value of the primary endpoint with 90% power using a two-sided 5% significance test in the primary analysis. Based on a consensus of expert opinion on the trial steering committee, a difference of 10% was thought to be of clinical relevance. Recruitment rates were lower than expected and an interim masked analysis was done in March, 2018, after 82 infants had been recruited. The revised estimate of the SD for the primary endpoint (16·6, 95% CI 14·0–20·6) suggested that the original sample size would provide 92·5% power, whereas concluding the study with 182 patients rather than the planned 200 would reduce the conservative estimate of power from 92·5% to 90%. The data monitoring and ethics committee and the trial steering committee decided that this would be an acceptable compromise to be able to report trial results within the desired timelines. The trial steering committee therefore recommended that the sponsor suspend recruitment when 182 infants had been recruited.

Analyses were predetermined in the statistical analysis plan to compare real-time CGM intervention with standard care. All analyses were done in the full analysis set with available data on an intention-to-treat basis. Linear regression was used to estimate the absolute difference in the proportion of time sensor glucose concentration in the target range of 2·6–10 mmol/L, adjusting for baseline variables (centre and gestational age). Secondary and exploratory endpoints that were continuous variables were analysed in the same way, and binary or ordinal variables were analysed using logistic or ordinal logistic regression.

To control the study-wide significance level to 5%, a Benjamini-Hochberg multiple testing procedure was applied to three endpoints of equal importance (proportion of time the sensor glucose concentration was 2·6–10 mmol/L, proportion of time the sensor glucose concentration was 4–8 mmol/L, and mean sensor glucose concentration). This was followed by a gate-keeping sequence applied to the remaining two variables, sensor glucose variability within individuals and proportion of time that sensor glucose concentrations were in the hyperglycaemic range (>15 mmol/L). Estimates of treatment effect, with 95% CIs and adjusted p values, were calculated. The primary and secondary efficacy analyses used all available sensor glucose measurements for each infant. Sensitivity analyses of the primary outcome were: linear regression adjusted for time to first sensor glucose measurement and the first sensor glucose value recorded; linear regression weighted by the number of CGM measurements per infant; generalised least squares regression allowing variance to differ between treatment groups; and a random effects model with a random intercept for site.

This study is registered with the International Standard Randomised Controlled Trial Registry, ISRCTN12793535.

### Role of the funding source

Neither the funders nor sponsor had any role in study design, data collection, data analysis, data interpretation, or writing of the report. Raw data was accessible to the trial statisticians and data team (SB, AA, BP, SK, SP). All authors had full access to all the data in the study and had final responsibility for the decision to submit for publication.

## Results

Between July 4, 2016, and Jan 27, 2019, 360 newborn infants deemed eligible on the basis of birthweight (≤1200 g) were identified within 24 h of birth, and 182 were enrolled in the trial after their parents consented, giving a consent rate of 51%. There was a short recruitment window and some families could not be approached because the parents were not available or there were no available research staff during the 24 h after birth (figure). 180 infants were randomly assigned, 85 to intervention with real-time CGM and 95 to standard care. There were 11 withdrawals from the trial and eight deaths (intervention n=2, standard care n=6); one infant in the CGM group was later found to be ineligible because they were older than 24 h and they were excluded from the study and analysis. Baseline characteristics of all infants eligible after randomisation are shown in table 1. CGM data were collected for 155 patients (intervention n=70, standard care n=85; distribution by centre shown in appendix p 7). Two infants were randomly assigned but were withdrawn before sensor insertion. The mean number of CGM data points in the real-time CGM intervention group was 1538 (SD 341) and in the standard care group was 1412 (424). The distribution of glucose concentrations recorded on the CGM devices in the two study groups is shown in appendix p 3.

**Figure:**
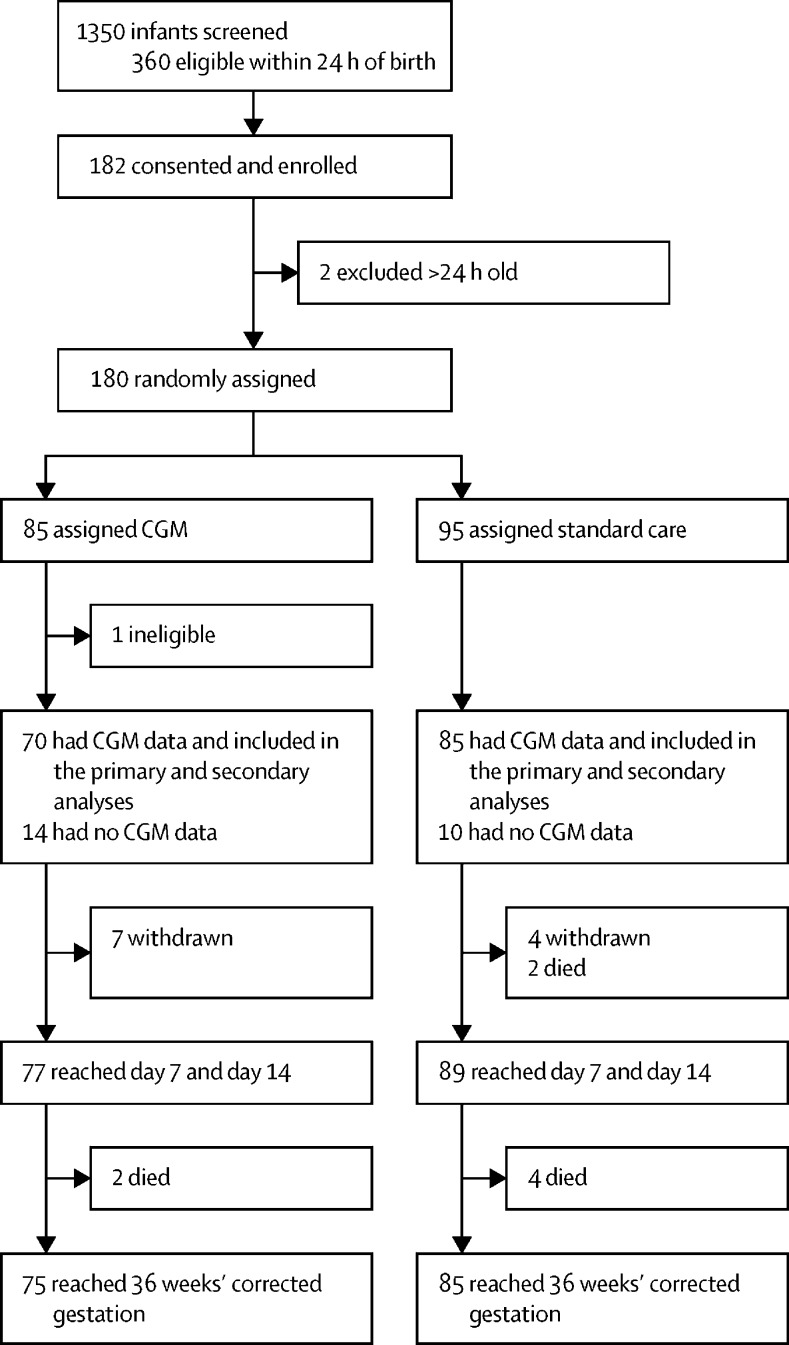
Trial profile All infants who were consented, with the exception of the three ineligible infants, were included in the safety analysis. CGM=continuous glucose monitoring.

**Table 1:** Baseline characteristics

		**CGM group (n=84)**	**Standard care group (n=95)**
Gestational age, weeks			
	Mean	27·7 (2·1)	27·4 (1·9)
	Range	24·0–33·7	23·3–31·3
Birthweight, g		910 (160)	880 (180)
Birthweight SD score		−0·82 (0·98)	−0·80 (0·92)
Sex			
	Male	45 (54%)	45 (47%)
	Female	39 (46%)	50 (53%)
Ethnicity*			
	Asian	7 (8%)	11 (12%)
	Black	7 (8%)	8 (8%)
	Mixed	6 (7%)	5 (5%)
	Other	3 (4%)	5 (5%)
	White	61 (73%)	66 (69%)
Number of infants delivered			
	Singleton	65 (77%)	70 (74%)
	Multiple	19 (23%)	25 (26%)
Mean CRIB II score†		10 (2·9)	10 (2·9)
Received antenatal steroids more than 24 h before delivery		69 (82%)	69 (73%)
Received antibiotics within 24 h of delivery		25 (30%)	32 (34%)
Maternal diabetes		5 (6%)	8 (8%)
Maternal chorioamnionitis		19 (23%)	21 (22%)
Delivery mode			
	Spontaneous vaginal delivery	29 (35%)	31 (33%)
	Caesarean section before onset of labour	41 (49%)	40 (42%)
	Caesarean section after onset of labour	13 (15%)	22 (23%)
	Assisted vaginal delivery	1 (1%)	2 (2%)

Data are mean (SD), range, or n (%). CGM=continuous glucose monitoring. CRIB=Clinical Risk Index for Babies.

* Ethnicity was parent-reported.

† For CRIB II score, data are available for n=91 in the standard care group and n=80 in the CGM group.

The primary outcome, proportion of time that sensor glucose concentrations were 2·6–10 mmol/L, was 94% (SD 11) in the real-time CGM group and 84% (22) in the standard care group (table 2). The intervention with real-time CGM compared with standard care increased the time that sensor glucose concentration was in the target range by 8·9 percentage points (95% CI 3·4–14·4; adjusted p=0·005). This is equivalent to an increase of 13 h (95% CI 5–21) within the target range during the first 6 days of life. Sensitivity analyses showed similar treatment effects, suggesting the results are robust (appendix pp 10–13).

**Table 2:** Primary and secondary efficacy outcomes

	**CGM group (n=70)**	**Standard care group (n=85)**	**Linear regression model**	
			Adjusted mean difference* (95% CI)	p value†
**Proportion of time sensor glucose was 2·6–10 mmol/L**				
Mean	94% (11)	84% (22)	8·9% (3·4 to 14·4)	0·005
Median	99% (92–100)	97% (71–100)	..	..
**Proportion of time sensor glucose was 4–8 mmol/L**				
Mean	74% (20)	62% (29)	11·5% (3·7 to 19·2)	0·006
Median	78% (61–89)	65% (39–88)	..	..
**Sensor glucose concentration, mmol/L**				
Mean	6·5 (1·2)	7 (2·1)	−0·46 (−1·00 to 0·09)	NA
Median	6·4 (5·8–9·9)	6·5 (5·2–8·4)	..	..
**Sensor glucose variability**				
Mean	2·7 (3·0)	3·6 (4·3)	−0·01 (−0·15 to 0·13)	NA
Median	1·9 (1·1–3·2)	2·1 (1·1–4·2)	..	..
**Proportion of time in hyperglycaemic range (sensor glucose >15 mmol/L)**				
Mean	0·3% (2·1)	1·3% (4·2)	−0·92% (−2·03 to 0·19)	NA
Median	0·0% (0·0–0·0)	0·0% (0·0–0·0)	..	..
**Proportion of time in hypoglycaemic range (sensor glucose <2·6 mmol/L)**				
Mean	1·0 (5·3)	1·1 (3·2)	−0·09 (−1·52 to 1·33)	NA
Median	0·0 (0·0–0·2)	0·0 (0·0–0·1)	..	..

Data are mean (SD) or median (IQR), unless indicated otherwise.

* Adjusted for gestation and centre.

† Adjusted for Benjamini-Hochberg test procedure. CGM=continuous glucose monitoring. NA=not applicable (due to the multiple testing procedure, p values are only reported up to the first non-significant p value).

Secondary outcome analyses showed an increase of 11·5 percentage points (95% CI 3·7–19·2; adjusted p=0·006) in the proportion of time in the target range of 4–8 mmol/L, equivalent to 17 h (95% CI 5–28), in the real-time CGM intervention group compared with the standard care group. Mean sensor glucose concentration, sensor glucose concentration variability, and the proportion of time that sensor glucose concentration was more than 15 mmol/L did not differ significantly between the two groups (table 2).

The results of the exploratory analyses are shown in table 3 and the appendix (p 8). More infants in the real-time CGM intervention group (49 [61%] of 80) received insulin in the first week of life than those in the standard care group (35 [37%] of 94); however, there was no significant difference in total insulin infused between the two groups (table 3). Infants in the real-time CGM intervention group received more infused daily glucose than infants in the standard care group (mean difference 0·69 g/kg; 95% CI 0·02–1·35). The incidence of necrotising enterocolitis was lower in the real-time CGM intervention group (10 [13%] of 75 infants) than in the standard care group (24 [28%] of 85; adjusted odds ratio [OR] 0·33; 95% CI 0·13–0·78); for the other exploratory clinical outcomes, no significant differences between groups were seen (appendix p 8).

**Table 3:** Drug and nutritional data in the first week of life

	**CGM group (n=80)**	**Standard care group (n=94)**	**Adjusted mean difference (95% CI)**	**p value**
**Medication received**				
Inotropes	19 (24%)	27 (29%)	..	..
Antibiotics	75 (94%)	90 (96%)	..	..
Caffeine	80 (100%)	94 (100%)	..	..
Morphine	32 (40%)	40 (43%)	..	..
Corticosteroids	3 (4%)	6 (6%)	..	..
Insulin	49 (61%)	35 (37%)	..	..
**Nutritional intake**				
Daily intravenous glucose, g/kg*	9·9 (2·6)	9·2 (2·4)	0·69 (0·02 to 1·35)	0·043
Daily intravenous amino acids, g/kg*	2·2 (1·2)	2·2 (1·3)	0·17 (−0·06 to 0·40)	0·58
Daily intravenous lipids, g/kg*	1·89 (0·77)	1·95 (0·78)	−0·04 (−0·21 to 0·13)	0·64
Daily oral nutrition, mL/kg*†	20 (21)	18 (23)	2·0 (−3·6 to 7·6)	0·49
Daily intravenous fluids, mL/kg*	118 (23)	113 (24)	NA	NA
Total insulin, units per kg per day*	0·60 (0·94)	0·57 (2·14)	0·08 (−3·3 to 3·5)	0·96

Data are n (%) or mean (SD), unless indicated otherwise. Mean differences were adjusted for gestational age and centre. CGM=continuous glucose monitoring. NA=not available: daily intravenous fluids were assessed post hoc and no statistical comparison was done.

* Data available for: daily glucose n=84 in the standard care group, n=81 in the GCM group; daily amino acids n=94 in the standard care group, n=81 in the CGM group; daily lipids n=92 in the standard care group, n=80 in the CGM group; daily oral nutrition n=94 in the standard care group, n=81 in the CGM group; daily intravenous fluids n=94 in the standard care group, n=81 in the CGM group; total insulin n=94 in the standard care group, n=80 in the CGM group.

† Oral nutrition included expressed maternal milk, donor milk, or preterm formula, dependent on availability and maternal preference.

There was no significant difference between the two groups for any of the safety analyses (appendix p 9): at least one episode of severe hypoglycaemia (blood glucose concentration of ≤2·2 mmol/L) was recorded in ten (13%) of 75 infants in the intervention group and in six (7%) of 93 infants in the standard care group; at least one episode blood glucose concentration of 2·2–2·6 mmol/L was recorded in 11 (15%) of 75 infants in the intervention group and in 11 (12%) of 93 infants in the standard care group. Among infants with CGM data, four (6%) of 70 in the intervention group and 13 (15%) of 85 in the standard care group a had at least one episode of sensor glucose concentration of less than 2·6 mmol/L for more than 1 h; mean total time with a sensor glucose concentration of less than 2·6 mmol/L was 0·5 h (SD 1·7) in the real-time CGM intervention group and 1·0 h (3·2) in the standard care group. There were no serious adverse events related to the use of the device or episodes of infection and the device did not appear to cause any discomfort.

Staff and parents reported that the real-time CGM did not interfere with the infants' care or cause any distress. Moreover, 35 (80%) of 44 parents and 74 (70%) of 106 staff reported that the use of CGM improved clinical care (appendix pp 4–5). Staff reported an increase in work load, although the free text included in the questionnaire suggested this related to the paperwork associated with trial data collection rather than the intervention per se. The time interval between blood glucose measurements did not differ between the two groups: mean 7·1 h (SD 4·7) in the intervention group versus 5·7 h (4·4) in the standard care group.

## Discussion

This multicentre study shows that intervention with real-time CGM, even outside centres with previous experience or special interest in the use of CGM, can improve glucose control in preterm infants. Compared with the standard care group, infants in the real-time CGM intervention group had reduced exposure to prolonged or severe hyperglycaemia and hypoglycaemia, which has implications for long-term health outcomes. Furthermore, staff and parents felt that the intervention improved care.

High energy and nutritional requirements and variable insulin sensitivity in preterm infants makes preventing hyperglycaemia, without the risk of hypoglycaemia, challenging. Although reducing dextrose infusion rates can ameliorate hyperglycaemia, glucose supply seems to have only a limited effect on blood glucose concentrations.^16^, ^22^ Insulin treatment can help to maintain normoglycaemia while supporting optimal nutritional delivery, and insulin treatment has been associated with lower mortality in infants with hyperglycaemia.^23^ However, substantial concern remains about the safety of insulin due to the risk of hypoglycaemia using current intermittent blood glucose monitoring. This study supports previous single-centre studies using real-time CGM in newborn infants but is unique in allowing clinicians to use the CGM data to optimise nutritional delivery as well as providing guidance on using insulin.^24^

This study also included the most preterm infants, as it is these infants who are most at risk of glucose dysregulation, and in whom improvements in glycaemic control could have the most impact on morbidity and mortality. We did exploratory analyses to determine the effects of improved glucose control in our cohort. Fewer babies in the real-time CGM intervention group died than in the control group but the study was not powered to show differences in mortality. Although the study was also underpowered to show differences in morbidity outcomes, one relevant outcome was the higher rate of necrotising enterocolitis in the standard care group than in the real-time CGM intervention group. Data have shown a causal pathway linking hyperglycaemia to an increased risk of microbial gut translocation in both animal models and adult studies.^25^ With necrotising enterocolitis being a major cause of mortality and morbidity this is a relevant finding, but larger studies would be needed to confirm the link. There is also good animal and human evidence to link hyperglycaemia with retinopathy of prematurity, bronchopulmonary dysplasia, and intraventricular haemorrhage and reduced white matter at term, but our exploratory analyses of these relationships were not significant.

Any benefits in terms of clinical outcomes might be related to glucose concentrations per se or to the resulting clinical decision-making and interventions. The increased time in target glucose concentration range in the intervention group was associated with a larger number of infants receiving insulin than in the standard care group, although there was no difference in the total amount of insulin infused between the groups. There was also a significant increase in amount of glucose infused in the intervention group, although this remained within the recommended range of glucose requirements for these infants. Together these findings suggest less insulin resistance in the infants in the real-time CGM group than in the standard care group. Reduced insulin resistance in the intervention group might be explained by the fact that hyperglycaemia induces insulin resistance,^26^ and reflects the clinical finding that infants who have developed persistent hyperglycaemia require higher doses of insulin to regain control, and might consequently be at higher risk of hypoglycaemia. Further studies will be needed to determine the optimal nutritional support and use of insulin in these infants, but the use of real-time CGM provides the opportunity for such interventions to be tested more safely.

As with previous studies, the use of masked CGM identified clinically silent episodes of hypoglycaemia.^10^, ^24^, ^27^ The CGM data showed numerically more babies in the standard care group being exposed to one or more episodes of hypoglycaemia for more than 1 h compared with those in the intervention group, although the study was not powered to detect a statistically significant difference. The mean length of time that sensor glucose was less than 2·6 mmol/L appeared longer in the standard care group than in the real-time CGM intervention group. Previous follow-up studies of infants with such clinically silent hypoglycaemia have shown these silent episodes to be associated with a dose-dependent increased risk of impaired developmental outcomes in childhood.^9^ The reduction in hyperglycaemia, which has been associated with increased risk of multiple neonatal morbidities as well as poor growth, also has the potential to improve childhood outcomes in the long-term.^28^

Clinical management in the intervention group was based on the ability to be guided by trends in glucose concentrations shown by the real-time CGM data and did not require blood glucose concentrations to be checked before altering treatment. However, the advice on checking blood glucose concentrations when there was a rapid change in CGM values or if CGM values fell to less than 4 mmol/L was key to ensuring a safe decision system and resulted in unanticipated hypoglycaemia being detected. This approach could have resulted in an increase in blood glucose measurements, but the time interval between point of care blood glucose measurement did not differ between the two groups.

The guideline approach (as shown in appendix p 2) within the trial meant that the intervention was dependent on clinical nursing staff engagement to take on board new technology, to act on the data, and to modify management 24 h a day. This makes such an intervention challenging to deliver and to show a clinical effect. This might be reflected in the staff questionnaire, particularly the comments relating to increased workload, although the more specific free text comments highlighted the workload related to completion of trial paperwork rather than the intervention itself. Despite these comments and some difficulties with hand insertion of the sensors, the staff and parents reported that the real-time CGM improved clinical care (appendix pp 4–5). Real-time CGM appeared to be safe for use in these preterm infants.

The limitations of this study include the potential recruitment bias resulting from a short recruitment window due to the desire to optimise early glucose control. This early control might be important if insulin resistance is to be minimised. There was also more data loss than in the feasibility study because of technical difficulties with sensor insertion, and because the protocol limited repeat insertions.^18^ Although the technique of hand insertion is not difficult and, like other procedures, becomes easier with experience, this might have affected the integrity of the sensor once in place and caused a consequent loss of data. Lessons learnt during the study in terms of techniques for insertion and response to alarms helped to optimise their use. Features designed for safety, because CGM devices are for personal, independent patient use, led to the need for some repeated calibrations and sensor shutting off and consequently potentially unnecessary sensor replacement. This highlights the potential benefits of bespoke technology for these preterm infants. Repeat insertion was limited by the protocol,^18^ which contrasts with similar clinical procedures that are often done more than once before being successful. It was not possible to mask the clinical or research teams to the study intervention, but compliance with masking of the CGM in the control group of the study was good. The point accuracy of CGM remains controversial but has been validated in newborn babies,^20^ and collecting data by CGM in both groups removed any bias associated with differences in the frequency of blood glucose sampling.

A further limitation is that this study was not powered to show the clinical effect of the difference in hyperglycaemia. However, this can be viewed in the context of previous studies that have shown that everyday exposure to hyperglycaemia (similar to the difference shown in this study) increased the risk of retinopathy of prematurity independent of the risks associated with decreasing gestational age and increasing immaturity (OR 1·073, 95% CI 1·004–1·146; p=0·04).^29^ In a large national cohort study (EXPRESS), Zamir and colleagues showed that the presence of hyperglycaemia more than doubled adjusted 28-day mortality (OR 2·55; p=0·005).^23^ Furthermore, treating infants who are hyperglycaemic with insulin was associated with lower 28-day mortality than not treating them (OR 0·15, 95% CI 0·04–0·53; p=0·003).^23^ Data from EXPRESS show the potential effect of not treating babies with hyperglycaemia, with those who were untreated for 1 day having more than 5 times the risk of mortality and those who were untreated for 2 days having more than 7 times the risk of mortality, compared with those who were treated.^23^

In summary, the use of CGM allowed earlier detection of and prevention of exposure to the extremes of both hypoglycaemia and hyperglycaemia in preterm infants. Lessons learnt during the trial will support advances in CGM for these babies. Further studies using CGM are required to determine optimal glucose targets, strategies to obtain them, and the potential effect on longer term health outcomes. The positive engagement of the clinical staff supports the potential for CGM to become an established part of clinical care as well as a valuable research tool.

## Data sharing

Anonymised data collected for the study, including individual participant data and a data dictionary defining each field in the set, will be made available 1 year after publication, after approval of a proposal and with a signed data access agreement. All data requests should be submitted to the corresponding author for consideration.

## References

[1] Garg R, Agthe AG, Donohue PK, Lehmann CU (2003). Hyperglycemia and retinopathy of prematurity in very low birth weight infants. J Perinatol.

[2] Kao LS, Morris BH, Lally KP, Stewart CD, Huseby V, Kennedy KA (2006). Hyperglycemia and morbidity and mortality in extremely low birth weight infants. J Perinatol.

[3] Hays SP, Smith EO, Sunehag AL (2006). Hyperglycemia is a risk factor for early death and morbidity in extremely low birth-weight infants. Pediatrics.

[4] Yager JY (2002). Hypoglycemic injury to the immature brain. Clin Perinatol.

[5] Finberg L (1967). Dangers to infants caused by changes in osmolal concentration. Pediatrics.

[6] Alexandrou G, Skiöld B, Karlén J (2010). Early hyperglycemia is a risk factor for death and white matter reduction in preterm infants. Pediatrics.

[7] van den Berghe G, Wouters P, Weekers F (2001). Intensive insulin therapy in critically ill patients. N Engl J Med.

[8] Beardsall K (2010). Measurement of glucose levels in the newborn. Early Hum Dev.

[9] McKinlay CJD, Alsweiler JM, Anstice NS (2017). Association of neonatal glycemia with neurodevelopmental outcomes at 4·5 years. JAMA Pediatr.

[10] Beardsall K, Vanhaesebrouck S, Ogilvy-Stuart AL (2008). Early insulin therapy in very-low-birth-weight infants. N Engl J Med.

[11] Leelarathna L, English SW, Thabit H (2014). Accuracy of subcutaneous continuous glucose monitoring in critically ill adults: improved sensor performance with enhanced calibrations. Diabetes Technol Ther.

[12] Bridges BC, Preissig CM, Maher KO, Rigby MR (2010). Continuous glucose monitors prove highly accurate in critically ill children. Crit Care.

[13] Battelino T, Danne T, Bergenstal RM (2019). Clinical targets for continuous glucose monitoring data interpretation: recommendations from the International Consensus on Time in Range. Diabetes Care.

[14] Wernerman J, Desaive T, Finfer S (2014). Continuous glucose control in the ICU: report of a 2013 round table meeting. Crit Care.

[15] Beardsall K, Ogilvy-Stuart AL, Ahluwalia J, Thompson M, Dunger DB (2005). The continuous glucose monitoring sensor in neonatal intensive care. Arch Dis Child Fetal Neonatal Ed.

[16] Beardsall K, Vanhaesebrouck S, Ogilvy-Stuart AL (2010). Prevalence and determinants of hyperglycemia in very low birth weight infants: cohort analyses of the NIRTURE study. J Pediatr.

[17] Beardsall K, Thomson L, Elleri D, Dunger DB, Hovorka R (2020). Feasibility of automated insulin delivery guided by continuous glucose monitoring in preterm infants. Arch Dis Child Fetal Neonatal Ed.

[18] Thomson L, Elleri D, Bond S, Howlett J, Dunger DB, Beardsall K (2019). Targeting glucose control in preterm infants: pilot studies of continuous glucose monitoring. Arch Dis Child Fetal Neonatal Ed.

[19] Beardsall K, Thomson L, Guy C (2018). Protocol of a randomised controlled trial of real-time continuous glucose monitoring in neonatal intensive care ‘REACT’. BMJ Open.

[20] Beardsall K, Vanhaesebrouck S, Ogilvy-Stuart AL (2013). Validation of the continuous glucose monitoring sensor in preterm infants. Arch Dis Child Fetal Neonatal Ed.

[21] Dixon KC, Ferris RL, Marikar D (2017). Definition and monitoring of neonatal hypoglycaemia: a nationwide survey of NHS England Neonatal Units. Arch Dis Child Fetal Neonatal Ed.

[22] Fernández-Martínez MDM, Gómez-Llorente JL, Momblán-Cabo J (2020). Monitoring the incidence, duration and distribution of hyperglycaemia in very-low-birth-weight newborns and identifying associated factors. J Perinat Med.

[23] Zamir I, Tornevi A, Abrahamsson T (2018). Hyperglycemia in extremely preterm infants—insulin treatment, mortality and nutrient intakes. J Pediatr.

[24] Galderisi A, Facchinetti A, Steil GM (2017). Continuous glucose monitoring in very preterm infants: a randomized controlled trial. Pediatrics.

[25] Thaiss CA, Levy M, Grosheva I (2018). Hyperglycemia drives intestinal barrier dysfunction and risk for enteric infection. Science.

[26] Massillon D, Barzilai N, Chen W, Hu M, Rossetti L (1996). Glucose regulates in vivo glucose-6-phosphatase gene expression in the liver of diabetic rats. J Biol Chem.

[27] Nally LM, Bondy N, Doiev J, Buckingham BA, Wilson DM (2019). A feasibility study to detect neonatal hypoglycemia in infants of diabetic mothers using real-time continuous glucose monitoring. Diabetes Technol Ther.

[28] Zamir I, Stoltz Sjöström E, Edstedt Bonamy AK, Mohlkert LA, Norman M, Domellöf M (2019). Postnatal nutritional intakes and hyperglycemia as determinants of blood pressure at 6·5 years of age in children born extremely preterm. Pediatr Res.

[29] Mohamed S, Murray JC, Dagle JM, Colaizy T (2013). Hyperglycemia as a risk factor for the development of retinopathy of prematurity. BMC Pediatr.

