# Supplementary material for: Real-time continuous glucose monitoring in preterm infants (REACT): an international, open-label, randomised controlled trial
Source: Lancet Child Adolesc Health. 2021 Apr;5(4):265–73. doi: 10.1016/S2352-4642(20)30367-9 (PMC7970623; doi:10.1016/S2352-4642(20)30367-9)
Supplement: Supplementary appendix [file mmc1.pdf]

THE LANCET
Child & Adolescent Health

Supplementary appendix

This appendix formed part of the original submission and has been peer reviewed. We post it as supplied by the authors.

Supplement to: Beardsall K, Thomson L, Guy C, et al. Real-time continuous glucose monitoring in preterm infants (REACT): an international, open-label, randomised controlled trial. *Lancet Child Adolesc Health* 2021; published online Feb 9. http://dx.doi.org/10.1016/S2352-4642(20)30367-9.

Supplementary Data

1. **eFigure 1: Paper guideline**
2. **eFigure 2: Histogram of the primary outcome by treatment group (n=179)**
3. **eFigure 3: Staff questionnaire of acceptability**
4. **eFigure 4: Parent questionnaire of acceptability.**
5. **eFigure 5: Mean glycaemia and mean insulin infused by treatment arm**

6. **eTable 1: Summary Data of Number of Subjects per centre**
7. **eTable 2: Exploratory analyses of clinical outcomes**
8. **eTable 3: Safety Analyses**
9. **eTable 4: Sensitivity Analyses for Primary outcome**

Sensor Glucose mmol/l	Falling	Stable	Rising
<2.6	**Check Blood Glucose**<br>Stop any Insulin & Check all lines<br>Give additional Dextrose<br>Consider starting 20% Dextrose at 1ml/kg/hr	**Check Blood Glucose**<br>Stop any Insulin & Check all lines<br>Give additional Dextrose<br>Consider starting 20% Dextrose at 1ml/kg/hr	**Check Blood Glucose**<br>Review infusions & check lines<br>Ensure Insulin is not running<br>Consider starting/increasing<br>20% Dextrose at 1ml/kg/hr
2.6-4.0	**Check Blood Glucose**<br>Stop any Insulin & Check all lines<br>Give additional Dextrose<br>Consider starting 20% Dextrose at 1ml/kg/hr	**Check Blood Glucose**<br>Stop any Insulin & Check all lines<br>Give additional Dextrose<br>Consider starting 20% Dextrose at 1ml/kg/hr	**Observe the rate of rise**<br>Review infusions & check lines<br>Ensure Insulin is not running<br>Consider need for additional Dextrose
Target Range<br>4.0 - 8.0	**IN TARGET**<br>If the rate of fall means you will be <4.0mmol/l within 1 hour consider reducing Insulin	**IN TARGET**	**IN TARGET**<br>Consider weaning any additional<br>20% Dextrose
8.0-10.0	**Observe the rate of fall**<br>Consider ***reducing*** Insulin infusion rate by 25%	Stop any additional 20% Dextrose<br>or<br>Start Insulin at 0.05 units/kg/hr<br>or<br>if Insulin is already running ***increase***<br>Insulin infusion rate by 50%	Stop any additional 20% Dextrose<br>or<br>Start Insulin at 0.05 units/kg/hr<br>or<br>if Insulin is already running ***increase***<br>Insulin infusion rate by 50%
10-15.0	**Observe the rate of fall**<br>Consider ***increasing*** Insulin infusion rate by 25%	Stop any additional 20% Dextrose<br>or<br>Start Insulin at 0.05 units/kg/hr<br>or<br>if Insulin is already running ***increase***<br>Insulin infusion rate by 50%	Stop any additional 20% Dextrose<br>or<br>Start Insulin at 0.05 units/kg/hr<br>or<br>if Insulin is already running ***increase***<br>Insulin infusion rate by 50%
> 15	**Observe the rate of fall**<br>Consider ***increasing*** Insulin infusion rate by 50%	Start Insulin at 0.05 units/kg/hr<br>**or**<br>consider ***increasing*** Insulin infusion rate<br>by 100% (that is: Double)<br>**Always check infusion lines if there is little or no response to an intervention**	Start Insulin at 0.05 units/kg/hr<br>**or**<br>consider ***increasing*** Insulin infusion rate<br>by 100% (that is: Double)<br>**Always check infusion lines if there is little or no response to an intervention**
CRITICAL<br>CONCERN<br>IN TARGET	Please remember continuous glucose sensor readings are provided to support clinical management.<br>They provide additional information on trends in glucose levels which should be used to guide the need for blood glucose measurement.<br>Capillary/venous blood glucose levels are more accurate.<br>***Always check infusion lines if there is little or no response to an intervention***		

Figure 1 Paper Guideline

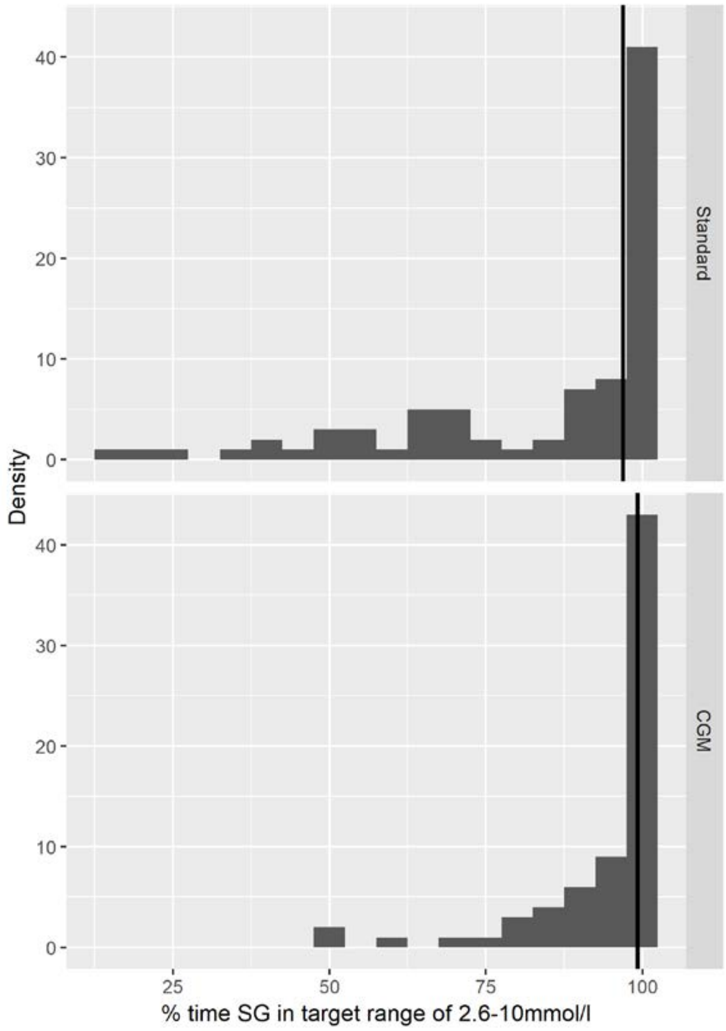

Figure 2 Histogram of the primary outcome by treatment group (n=179)

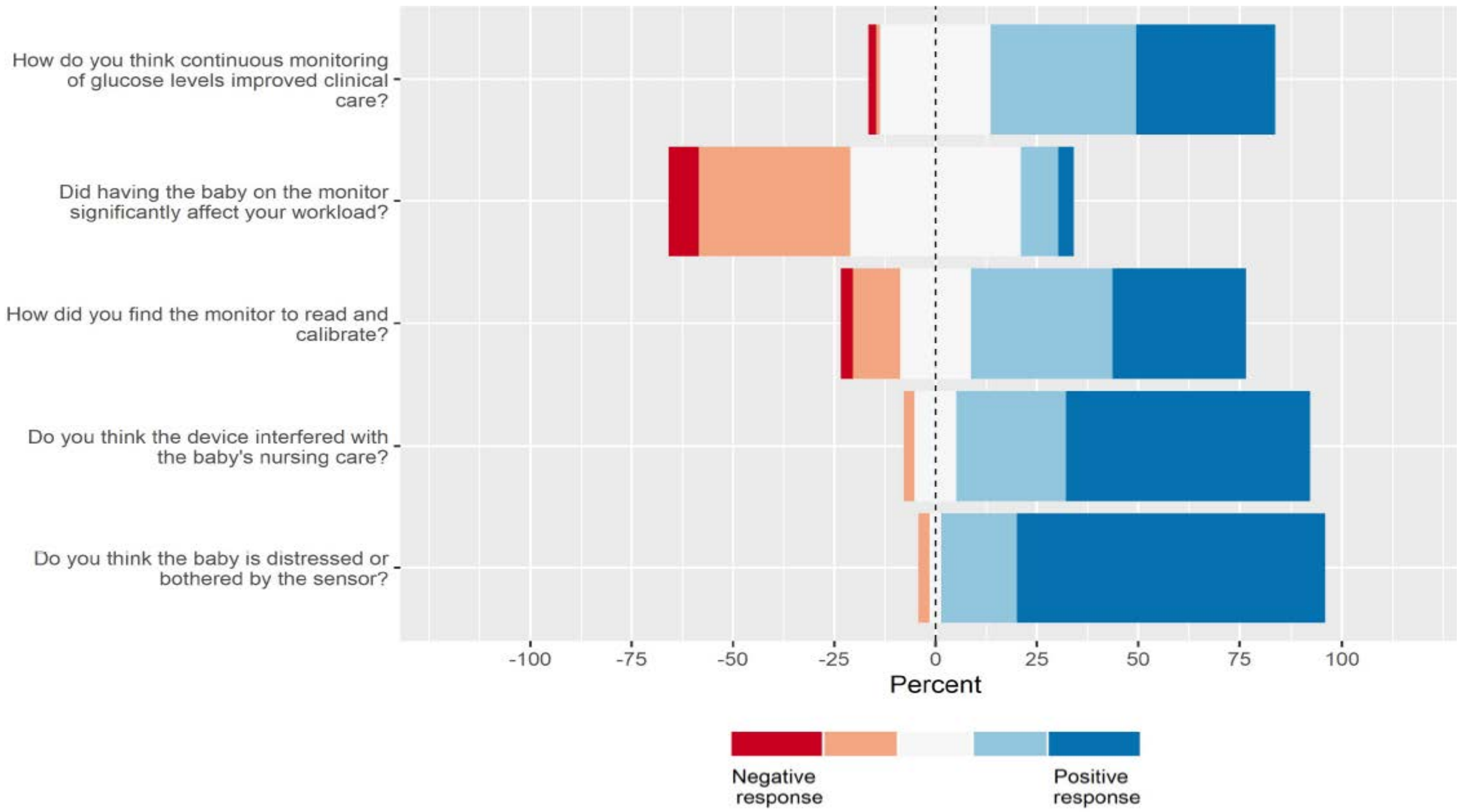

Figure 3 Staff Questionnaire of acceptability.

Stacked bar chart showing proportion of response for each Likert question Pooled data from day 3 and day 7 (n=54).

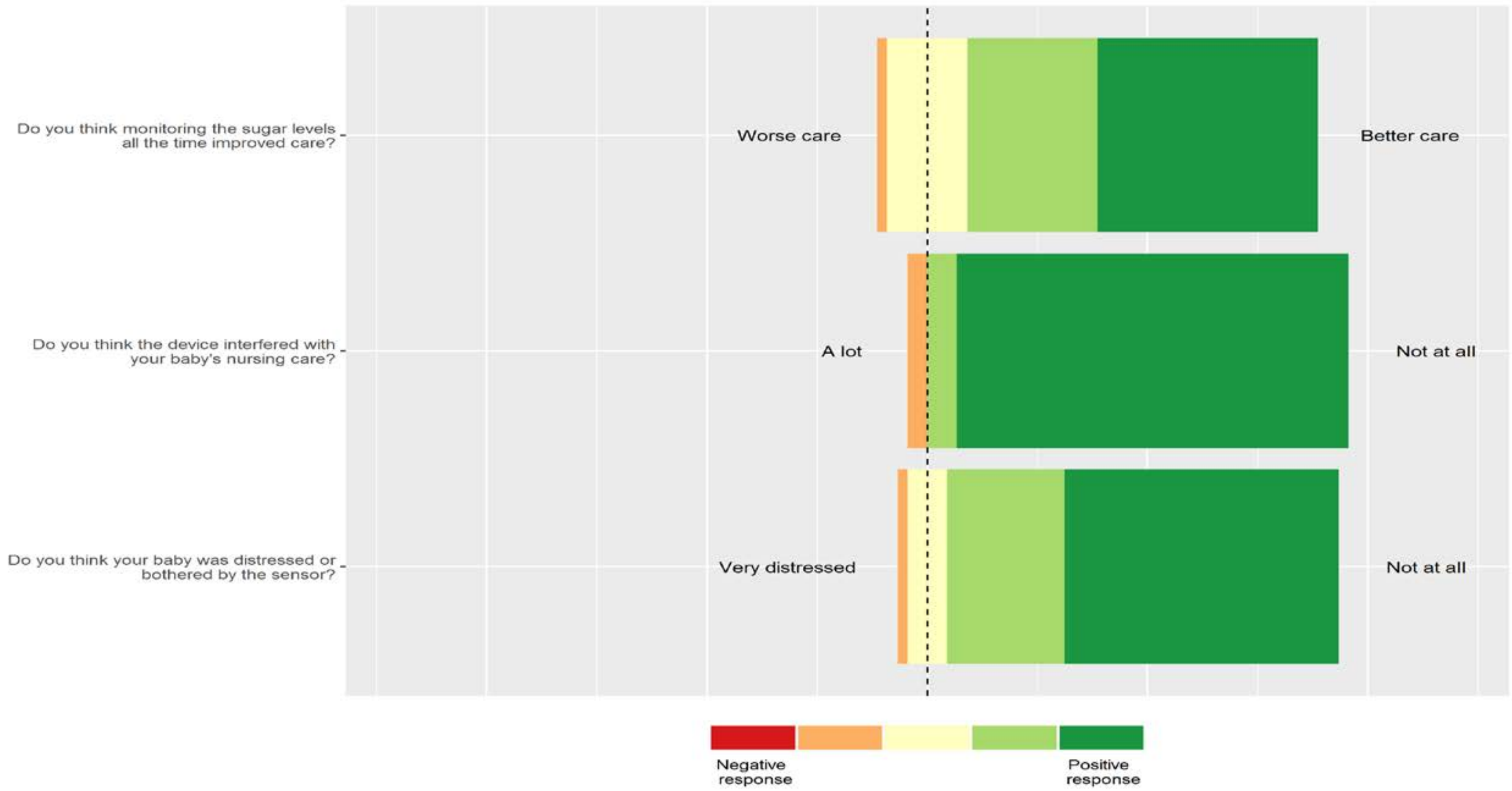

Figure 4 Parent Questionnaire of acceptability.

Stacked bar chart showing proportion of response for each Likert question (n=45)

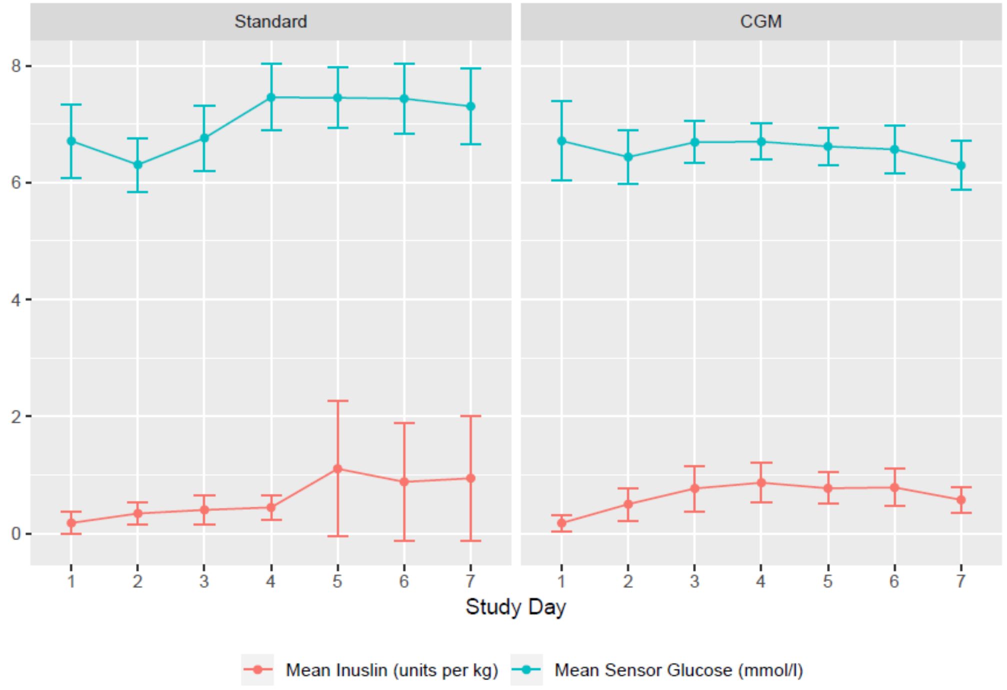

Figure 5 Mean glycaemia and mean insulin infused by treatment arm

CGM: Continuous glucose monitoring intervention

	mean	sd	median	Min	maz
**Standard Care**	**7.31**	**5**	**7.19**	**2**	**28**
**Real Time CGM**	**6.46**	**3**	**7.62**	**1**	**28**

Sites were adjusted for using a fixed effects model in the primary analysis. This was borderline case as to whether fixed- or random-effects for site were more appropriate. We report based on the pre-specified choice. Sensitivity analysis show the treatment effect is consistent with either modelling assumtpion: 9.26 (2.79) [estimate (SE)] in the random effects model

eTable 1. Summary Data of Number of Subjects per Centre

Outcome	Standard	CGM	Adjusted Estimate[a] (95% CI)	P-value
Mortality	6% (6/95)	2% (2/84)	0·263 [0·0353, 1·3]	0·13
Infection	62% (53/85)	58% (50/74)	1 [0·46, 2·2]	0·99
NEC	28% (24/85)	13% (10/75)	0·328 [0·129, 0·78]	0·014
PDA	31% (26/85)	26% (19/74)	0·52 [0·199, 1·3]	0·16
Intracerebral pathology	32% (27/84)	33% (25/75)	1·02 [0·51, 2·1]	0·95
BPD	66% (56/85)	60% (45/75)	1·2 [0·52, 2·8]	0·66
Maximum ROP	1·5 (3·6)	1·1 (3·8)	-0·26 (-1·37, 0·85)	0·64
Weight SDS – day 7	-1·3 (0·75)	-1·26 (0·79)	0·05 (-0·19, 0·28)	0·69
Weight SDS – 36 weeks gestation	-1·54 (0·94)	-1·56 (0·93)	-0·04 (-0·34, 0·25)	0·78
Body length SDS – day 7	-1·81 (1·07)	-1·78 (0·87)	-0·02 (-0·36, 0·31)	0·89
Body length SDS – 36 weeks gestation	-2·6 (1·6)	-2·9 (1·8)	-0·38 (-1·19, 0·42)	0·34
Head circumference – day 7	-1·84 (0·77)	-1·76 (0·83)	0·07 (-0·2, 0·35)	0·60
Head circumference – 36 weeks gestation	-1·3 (1·6)	-1·1 (1·7)	0·13 (-0·44, 0·7)	0·65
Total insulin – week 2 (units per kg)	1·1 (2·6)	1·1 (3·0)	0·1 (-0·76, 0·96)	0·82

Data presented as mean (SD) or percentage (frequency). [a]Adjusted for gestation and centre: data presented as mean difference (95% CI) and odds ratio [95% CI]. NEC: necrotizing enterocolitis; PDA: patent ductus arteriosus; BPD: bronchopulmonary dysplasia; ROP: retinopathy of prematurity; SDS: standard deviation score

eTable 2 Clinical Outcomes Exploratory Analyses

Variable	Statistics	Standard	CGM	Logistic regression	
				Adjusted Odds Ratio[a] (95% CI)	P-value
2.2mmol/l< BG <2.6mmol/l	Yes	11.8% (11/93)	14.7% (11/75)	1.2 (0.48, 3.2)	0.7
BG ≤2.2mmol/l	Yes	6.5% (6/93)	13.3% (10/75)	2.2 (0.7, 7.1)	0.2
Continuous episode of SG <2.6mmol/l for >1 hr	Yes	15.3% (13/85)	5.7% (4/70)	0.361 (0.0919, 1.2)	0.1
Length of time SG <2.6mmol/l (hours)	n	85	70		
	Mean (SD)	1.0 (3.2)	0.5 (1.7)		
	Median	0	0		
	Min, Max	0, 22	0, 11.2		
% time SG <2.6mmol/l	n	85	70		
	Mean (SD)	1.1 (3.2)	1 (5.3)		
	Median	0	0		
	Min, Max	0, 16.7	0, 41		

[a]Adjusted for centre and gestation (<26 weeks, ≥26 weeks)

eTable 3 Safety analyses of real time continuous glucose monitoring versus standard care

Title: Primary outcome
Population: Full Analysis (N = 179)
Subtitle: Model fitting results - sensitivity analyses

Model	Outcome	Covariate	Estimate (Std. Error)	95% CI	P-value
LM weighted by number of SG observations	% time SG in target of 2.6-10mmol/l	(Intercept)	80 (3.39)	(73.3, 86.7)	<0.001
		CGM versus standard	8.5 (2.58)	(3.4, 13.6)	0.001
		N36 (ref: N01)	-4.57 (15.1)	(-34.4, 25.3)	0.763
		N42	7.04 (5.5)	(-3.85, 17.9)	0.203
		N43	-0.0351 (3.82)	(-7.59, 7.52)	0.993
		N68	1.73 (3.95)	(-6.09, 9.54)	0.663
		N73	3.55 (5.39)	(-7.1, 14.2)	0.511
		N74	10 (12.4)	(-14.6, 34.6)	0.422
		N85	8.28 (6.43)	(-4.42, 21)	0.2
		N86	-21 (8.66)	(-38.1, -3.89)	0.017
		N87	-16.5 (12)	(-40.3, 7.31)	0.173
		N88	-26.5 (7.21)	(-40.8, -12.2)	<0.001
		ND3	6.33 (6.22)	(-5.96, 18.6)	0.311
		SP3	9.19 (7.64)	(-5.91, 24.3)	0.231
		Gestation >= 26 weeks	6.07 (3.29)	(-0.431, 12.6)	0.067
RSD = 15.8					
LM adjusted for start time of first SG and first SG	% time SG in target of 2.6-10mmol/l	(Intercept)	82.8 (4.66)	(73.6, 92)	<0.001
		CGM versus standard	9.09 (2.64)	(3.86, 14.3)	<0.001
		Time from birth (hours) to first SG	0.0353 (0.132)	(-0.226, 0.297)	0.79
		First SG measurement (mmol/l)	-2.3 (0.553)	(-3.4, -1.21)	<0.001
		N36 (ref: N01)	-12.8 (12.3)	(-37.1, 11.6)	0.302
		N42	2.77 (6.01)	(-9.11, 14.6)	0.645
		N43	0.00483 (4.03)	(-7.97, 7.98)	0.999
		N68	-2.76 (4.17)	(-11, 5.49)	0.51
		N73	-0.444 (5.29)	(-10.9, 10)	0.933

Model	Outcome	Covariate	Estimate (Std. Error)	95% CI	P-value
		N74	3.84 (12.2)	(-20.2, 27.9)	0.753
		N85	4.94 (6.61)	(-8.13, 18)	0.456
		N86	-14.6 (8.54)	(-31.4, 2.31)	0.09
		N87	-16.4 (11.9)	(-39.9, 7.16)	0.171
		N88	-26.9 (7.74)	(-42.2, -11.6)	<0.001
		ND3	2.7 (6.72)	(-10.6, 16)	0.688
		SP3	7.13 (8.46)	(-9.59, 23.9)	0.401
		Gestation >= 26 weeks	1.25 (3.51)	(-5.68, 8.18)	0.721
RSD = 16.14					
LM with interaction between time to first SG and treatment	% time SG in target of 2.6-10mmol/l	(Intercept)	82.6 (5.71)	(71.3, 93.9)	<0.001
		CGM versus standard	9.47 (6.83)	(-4.04, 23)	0.168
		Time from birth (hours) to first SG	0.043 (0.183)	(-0.319, 0.405)	0.815
		First SG measurement (mmol/l)	-2.3 (0.555)	(-3.4, -1.2)	<0.001
		N36 (ref: N01)	-12.8 (12.4)	(-37.4, 11.7)	0.303
		N42	2.71 (6.1)	(-9.35, 14.8)	0.657
		N43	-0.00716 (4.05)	(-8.02, 8.01)	0.999
		N68	-2.76 (4.19)	(-11, 5.52)	0.51
		N73	-0.467 (5.32)	(-11, 10.1)	0.93
		N74	3.96 (12.4)	(-20.5, 28.5)	0.749
		N85	4.93 (6.63)	(-8.18, 18)	0.458
		N86	-14.6 (8.62)	(-31.7, 2.42)	0.092
		N87	-16.5 (12)	(-40.3, 7.33)	0.173
		N88	-27 (7.97)	(-42.7, -11.2)	<0.001
		ND3	2.7 (6.75)	(-10.6, 16)	0.689
		SP3	7.11 (8.49)	(-9.68, 23.9)	0.404
		Gestation >= 26 weeks	1.26 (3.52)	(-5.7, 8.23)	0.72
		[CGM - treatment:Time from birth (hours) to first SG]	-0.0147 (0.242)	(-0.494, 0.465)	0.952
RSD = 16.2					

Model	Outcome	Covariate	Estimate (Std. Error)	95% CI	P-value
LM with interaction between first SG and treatment	% time SG in target of 2.6-10mmol/l	(Intercept)	80 (4.63)	(70.8, 89.1)	<0.001
		CGM versus standard	10.3 (2.61)	(5.17, 15.5)	<0.001
		First SG measurement (mmol/l)	-3.49 (0.673)	(-4.82, -2.16)	<0.001
		Time from birth (hours) to first SG	0.0591 (0.129)	(-0.196, 0.314)	0.648
		N36 (ref: N01)	-11.6 (12)	(-35.3, 12.2)	0.337
		N42	1.84 (5.86)	(-9.75, 13.4)	0.754
		N43	0.839 (3.94)	(-6.95, 8.63)	0.831
		N68	-2.56 (4.06)	(-10.6, 5.47)	0.53
		N73	-0.185 (5.15)	(-10.4, 10)	0.971
		N74	2.68 (11.9)	(-20.8, 26.1)	0.822
		N85	4.75 (6.43)	(-7.97, 17.5)	0.461
		N86	-19.1 (8.46)	(-35.9, -2.42)	0.025
		N87	-16.8 (11.6)	(-39.7, 6.12)	0.149
		N88	-27.1 (7.54)	(-42, -12.2)	<0.001
		ND3	3.11 (6.54)	(-9.83, 16.1)	0.635
		SP3	5.63 (8.25)	(-10.7, 21.9)	0.496
		Gestation >= 26 weeks	3.38 (3.49)	(-3.52, 10.3)	0.334
		[CGM - treatment:First SG measurement (mmol/l)]	3.07 (1.04)	(1, 5.13)	0.004
RSD = 15.71					
GLS allowing for heteroscedastic variances between treatment groups	% time SG in target of 2.6-10mmol/l	(Intercept)	81.9 (3.44)	(75.2, 88.7)	<0.001
		CGM versus standard	8.92 (2.7)	(3.63, 14.2)	0.001
		N36 (ref: N01)	2.88 (9.38)	(-15.5, 21.3)	0.759
		N42	8.28 (5.15)	(-1.82, 18.4)	0.11
		N43	3.87 (3.3)	(-2.59, 10.3)	0.242
		N68	-0.0845 (3.22)	(-6.4, 6.23)	0.979
		N73	4.67 (4.24)	(-3.63, 13)	0.272

Model	Outcome	Covariate	Estimate (Std. Error)	95% CI	P-value
		N74	9.2 (9.38)	(-9.18, 27.6)	0.328
		N85	8.46 (5.5)	(-2.33, 19.2)	0.126
		N86	-23.2 (8.15)	(-39.2, -7.26)	0.005
		N87	-11.8 (15.7)	(-42.5, 18.9)	0.453
		N88	-12.2 (6.5)	(-25, 0.523)	0.062
		ND3	7.3 (5.5)	(-3.48, 18.1)	0.187
		SP3	9.02 (6.79)	(-4.28, 22.3)	0.186
		Gestation >= 26 weeks	1.53 (2.93)	(-4.21, 7.27)	0.603
RSD = 9.59					
Random intercept for site	% time SG in target of 2.6-10mmol/l	(Intercept)	79.5 (4.14)	(71.3, 87.7)	<0.001
		CGM versus standard	9.26 (2.79)	(3.75, 14.8)	0.001
		Gestation >= 26 weeks	5.19 (3.56)	(-1.84, 12.2)	0.146
RSD = 17.15					
LM adjusting for gestation only	% time SG in target of 2.6-10mmol/l	(Intercept)	80.1 (3.45)	(73.3, 86.9)	<0.001
		CGM versus standard	9.38 (2.88)	(3.7, 15.1)	0.001
		Gestation >= 26 weeks	5.28 (3.62)	(-1.87, 12.4)	0.147
RSD = 17.8					

RSD = residual error standard deviation; LM = linear regression model; GLS = generalised least squares

eTable 4: Sensitivity Analyses for Primary outcome
